# Medical and Physical Intelligence

**Published:** 1820-12

**Authors:** 


### Medical and Physical Intelligence.

*Copy of the Report to the Secretary of State for the Home Department, from the National Vaccine Establishment, dated 18th May, 1820.*

To the Right Honourable LORD VISCOUNT SIDMOUTH, Principal Secretary of State for the Home Department, &c. &c. &c.

*National Vaccine Establishment, Percy-street; 18th May, 1820.*

MY LORD,

THE Board of the National Vaccine Establishment have the honour to report to your Lordship, that the number of persons vaccinated during the last year, in London and its vicinity, exceeds the number of any former year; it amounts to 8,957. Within the same year, 51,005 charges of vaccine lymph have been distributed to the public.

An abundant, an unceasing supply, which could only be afforded by such an Institution as that which the Board have the honour to direct, has enabled us to answer the earnest demands for vaccine lymph, from various parts of Great Britain and Ireland, from Jamaica, St. Vincent's, Dominica, Tortola, Grenada, Nevis, Montserrat, Antigua, St. Christopher's, Demerara, Hayti, and the Cape of Good Hope.

Lymph has also been occasionally requested from the continent of Europe, and charges were lately transmitted to Hamburgh and Hanover.

Our correspondents in Great Britain and Ireland have reported to this Board, that the number of persons vaccinated by them, during the year 1819, amounts to 74,940; forming, with the number vaccinated in London and its vicinity, a total of 83,897 persons in one year: yet many send no returns, or the number would be considerably greater.

From these facts the Board think themselves entitled to conclude, that the practice of vaccination in his Majesty's dominions continues to advance, and therefore that the confidence of medical practitioners, and the confidence of the public in that practice, remain unshaken; notwithstanding many unfavourable occurrences, with which it will be our duty to acquaint your Lordship.

The reports transmitted to this Board likewise warrant the conclusion, that, wherever small-pox inoculation is abandoned, and vaccination exclusively favoured or commanded, the most striking illustrations of the value of the Jennerian discovery are uniformly afforded; for, in addition to those places mentioned in former reports, in which small-pox is now unknown, the Board have received information that no case of that disease has occurred since the year 1804, at Shottisham in Norfolk; nor since the year 1817, in the city of Gloucester. The boroughs of Clonmell and Newton Limavady in Ireland, and Mothvey in Carmarthenshire, with the whole country for twenty miles around it, are reported to have completely succeeded in the extirpation of the small-pox; and in the island of Guernsey, only one solitary case of that fatal distemper is known to have occurred during the last year.

The career of vaccination appears, however, to have been less brilliant in its native country than in some parts of the continent of Europe, where the practice of it is enforced by legal enactments, and inoculation for small-pox is prohibited by severe penalties. Under such regulations, it is affirmed that the small-pox has ceased to exist in Denmark for the last eight years; and that the knowledge of this fact has now induced his Danish majesty to proclaim the same decrees in his West-India colonies.

The Board are also informed, by a most interesting communication from Dr. De Carro of Vienna, that similar decrees have been published in the Austrian dominions, and that small-pox is now confined to that portion of the poor who, by concealment, contrive to evade the imperial ordinances. He announces that, since the year 1799, when he gave the first example to the continent of Europe by vaccinating his two elder sons, he has never seen a single case to weaken his confidence in the efficacy of that practice.

An important letter, together with a treatise on this subject, has also been transmitted to the Board from Dr. Krauss, an intelligent physician, who is charged with the superintendance of vaccination in the circle of Rezat in Bavaria. He affirms that in that circle, containing half a million of people, small-pox has never occurred since the year 1807.

If these facts be correctly reported to us, they would appear to afford convincing proof that the extinction of small-pox is entirely within our power.

The testimonies of some of our correspondents in this country, are by no means so favourable. They concur in showing, that great numbers of persons who had been vaccinated have been subsequently seized with a disease presenting all the essential characters of small-pox; but that, in the great majority of such cases, the disease has been of comparatively short duration, unattended by symptoms of danger. In several of these cases, however, the malady has been prolonged to its ordinary period; and in eight reported cases it has proved fatal.

It appears to us to be fairly established, that the disposition in the vaccinated to be thus affected by the contagion of small-pox, does not depend on the time that has elapsed after vaccination; since some persons have been so affected who had recently been vaccinated; whilst others, who had been vaccinated eighteen and twenty years, have been inoculated and fairly exposed to the same contagion with impunity.

Nor is it undeserving of remark, that, whilst cases of small-pox in the vaccinated have frequently been reported to us from some parts of the kingdom remote from the metropolis, no cases of a similar nature are known to have happened in other districts equally populous. Very intelligent surgeons in the different counties of Norfolk, Devonshire, Middlesex, Cheshire, and Staffordshire, who together have vaccinated more than 30,000 persons, assert that they never saw or heard of small-pox in any one of their vaccinated patients.

But no assertions of individuals, however respectable, are so well calculated to direct the judgment of your Lordship as the registers of public charities.

The practice of vaccination was begun in the Small-pox Hospital of London in the year 1799, soon after the promulgation of Dr. Jenner's discovery, and has been continued to the present day. In the last annual report it is stated by Dr. Ashburner, "that the benefit of vaccination has been extended within the year to 3,297 persons; that one only of the 46,662 cases mentioned in former reports has been since affected with the varioloid eruption occurring after vaccination."

At the Foundling Hospital, vaccination was introduced nineteen years ago; and we are informed by Dr. Stanger, that only two cases of disease, bearing any resemblance to small-pox, have hitherto occurred in the vaccinated of that institution.

Mr. Mac Gregor assures us, that, in the great assemblage of the sons and daughters of soldiers who are brought up at the Royal Military Asylum, no case, even of the mildest small-pox, has ever occurred after vaccination.

Under the immediate direction of the National Vaccine Establishment, more than 60,000 persons have now been vaccinated in London and its vicinity, and of this large number only five are reported to have been subsequently affected with small-pox; although positive orders are given at every station to report all such cases as are even suspected.

This success in London, where the vaccinated are continually exposed to the contagion of small-pox, is strong evidence in favour of the practice adopted and inculcated by this Board, and induces us to believe that a departure from that practice is one source of the evil which has prevailed in different parts of the kingdom.

The great principle of that practice is to affect the constitution of each individual very completely with the vaccine disease; and the board have thought it right to direct that lymph should never be employed from any vesicle in which the slightest irregularity or imperfection can be observed, nor even from a perfect vesicle after the areola is formed; that two punctures be made in each arm, in order to secure at least three perfect vesicles; that one vesicle on each arm should be left unopened, and the lymph be suffered to be absorbed or desiccate; that if the vesicles be accidently broken or much injured, or if they present any irregularity, the patient should be carefully re-vaccinated as at first.

From extensive experience and numerous reports, the Board have become most earnestly desirous that more, rather than fewer, vesicles should be produced. We think it especially wrong to confide in one vesicle, and highly imprudent to open all: but no treatment will be effective in certain constitutions; for twenty-one cases of small-pox occurring after small-pox have been reported to us within the last twelve months, three of which were fatal.

We have regarded it, my Lord, as one of our first duties to consider attentively the different cases of small-pox after vaccination, as they have been transmitted to us. We have endeavoured to investigate them, free from the influence of theory, and solely intent on the discovery of truth: and, when we take into our view the immense number of the vaccinated, when compared with the reported failures;—when we reflect on certain peculiarities of constitution, that will exempt some individuals from all common laws;—when we think on the ignorance and carelessness which the vaccinator has but too often betrayed;—when we recollect the mild form which small-pox is reported to have very generally, though not universally, assumed in the vaccinated;—we cannot hesitate to assert, that our conviction in favour of the experiment of universal vaccination is unshaken.

It is a painful duty for us to state to your Lordship, that 712 persons are reported, by the Bills of Mortality of London, to have died of small-pox within the last year; and that the ravages committed by this disease in many other cities, and in many parts of the country, have also been great; yet we believe them to be fairly attributable to the neglect of universal vaccination, and the partial but too-frequent practice of small-pox inoculation.

(Signed) J. Latham, m.d. *President.*

Arthur Daniel Stone,<br>Robert Bree,<br>Edward Thomas Munro,<br>Geo. L. Tuthill, } *Censors of the Royal College of Physicians.*

David Dundas, *Master of the Royal College of Surgeons.*

Thompson Forster,<br>Everard Home, } *Governors.*

By order of the Board,<br>James Hervey, m.d. *Registrar.*

---

The lists of the supporters of our public Medical Charities present such a proportion of the names of medical men, as show that they must feel greatly interested in the success of institutions of this kind; and it is probable that every practitioner occasionally meets with individuals whom he would wish to consign to some public charity, where they may receive *medicines* as well as advice gratuitously. It should not, however, be expected that medical men, who devote much of their time, in private, to charitable objects, can also generally contribute pecuniary aid to public Institutions; it will therefore be a source of gratification to those who practise in London to know that an Infirmary has been lately established here, for one class of patients, the regulations and objects of which will permit them to point out a source of medical aid to those who may require it, without any pecuniary contributions on their part being essentially necessary. On the 26th of April last, a meeting took place at St. Martin's Library, at which were present the Right Rev. the Lord Bishop of Chester, the Hon. and Right Rev. the Lord Bishop of Gloucester, Sir Abraham Hume, Bart. the Rev. A. Hamilton, Henry Holland, Esq. and several other gentlemen residing in different parishes within the city and liberties of

Westminster; when the following resolutions were past, amongst others, for the internal regulation of the charity.

"That it is expedient to establish a charitable institution, for the purpose of affording medical and surgical relief to the children of the poor under twelve years of age, throughout the western part of the metropolis.

"That, should the funds of the Institution hereafter admit of an extension of its advantages, a number of children, requiring constant attendance or surgical operations, be admitted in-patients.

"That vaccination be another object of the Institution.

"That all children of the poor resident within the city of Westminster and the parish of Marylebone, be admitted patients WITHOUT LETTERS OF RECOMMENDATION."

Two stations were soon afterwards opened, in conformity with those Resolutions, one in East-street, Manchester-square, the other in Barton-street, Westminster, for out-patients; the establishment of a central station, or that for receiving in-patients, being postponed until the funds of the Institution would permit it to be done in an effective manner. A Resolution to put this in force without delay, was, however, passed at the first quarterly General Court of the Directors and Governors, held on the 15th of November, the Right Rev. the Lord Bishop of Chester in the chair; when a letter was read from Sir B. Bloomfield, reporting that his Majesty had most graciously condescended to become the Patron of the Institution. An answer was also received from his Royal and Serene Highness Prince Leopold of Saxe-Cobourg, Vice-Patron of the Institution, through the Right Reverend the Chairman, to an application made at the last general meeting; upon which it was unanimously resolved, "That the charity be henceforward styled THE ROYAL METROPOLITAN INFIRMARY FOR SICK CHILDREN, under the patronage of his Majesty the King, consecrated to the memory of the late Princess Charlotte of Wales."

The propriety of this change in the title of the Institution was suggested by the committee, in order that this charity might be well distinguished by its appellation, as it is by its plan and regulations, from any other established in London. The only other Institution having the same express objects, is the Universal Dispensary, at St. Andrew's Hill, Doctors' Commons, (in the City;) and it appears from the Reports of that Institution, that only one hundred and fifty of the poor from the several parishes of Westminster have been relieved by it during the period of three years and a half; giving a proportion of about ten for the parishes of St. James, St. George, St. Ann, &c. collectively, for a space of eight weeks; whilst, during the same time, the Royal Infirmary, scarcely yet generally known to exist, has afforded medical aid to above two hundred. The utility of an Institution in the midst of the parishes just mentioned, and in that of Marylebone, *still more remote from the City,* became sufficiently obvious, independently of the advantages in the plan of the Infirmary, which does not require for the admission of patients any letters of recommendation; the trouble and difficulty of getting which prevents, in a great many instances, the benefits of such a charity being conferred when they are most requisite: as in cases of immediate danger, and when the parents of sick children cannot waste whole days (as is often necessary) in seeking for letters of this kind.

---

*Cantharadin.*—It appears from a set of experiments made by Dr. J. Freeman Dana, of which he gives an account in Silliman's American Journal of Science and the Arts, vol. ii. p. 137, that the *lytta vittata*, or common potatoe-fly of North America, contains a quantity of cantharadin as well as the Meloe vesicatoria. The lytta of America, Dr. Dana informs us, possesses vesicating powers in a higher degree than the Spanish fly. The experiments were made on rather a small scale, in consequence of the difficulty of procuring the fly in sufficient quantity; but a sensible portion of cantharadin seems to have been obtained.—*Annals of Philosophy.*

---

### MONTHLY CATALOGUE OF MEDICAL BOOKS.

### METEOROLOGICAL JOURNAL.

By Messrs. William Harris and Co. 50, Holborn, London.

*From October* 20 *to November* 19, *inclusive.*

| Day of Month. | Moon. | Rain gauge | THERM. | | | BAROM. | | DeLuc's Hygro. | | WIND. | | ATMOSPHERIC VARIATION. | | |
|---|---|---|---|---|---|---|---|---|---|---|---|---|---|---|
| Oct. | | | | | | | | | | | | | | |
| 20 | | | 45 | 50 | 38 | 29·17 | 29·33 | 55 | 58 | W | NW | Fine | | |
| 21 | ○ | | 43 | 49 | 39 | 29·45 | 29·57 | 58 | 60 | WNW | WSW | Fine | | |
| 22 | | ‘28 | 45 | 52 | 41 | 29·92 | 29·85 | 62 | 60 | WSW | WSW | Rain | | Cloudy |
| 23 | | ‘11 | 45 | 50 | 40 | 29·21 | 29·05 | 59 | 59 | SW | S | Cloudy | | |
| 24 | | ‘23 | 43 | 53 | 42 | 28·97 | 28·84 | 60 | 62 | SW | WSW | Cloudy | Rain | Cloudy |
| 25 | | ‘19 | 47 | 52 | 40 | 28·67 | 28·90 | 61 | 60 | WSW | SW | Rain | | Cloudy |
| 26 | | ‘07 | 45 | 54 | 43 | 29·34 | 29·03 | 59 | 60 | SSW | SW | Fog | Show’ry | Cloudy |
| 27 | | ‘08 | 50 | 56 | 37 | 29·07 | 29·46 | 60 | 57 | SW | NW | Cloudy | Rain | Cloudy |
| 28 | ☽ | | 42 | 57 | 39 | 29·60 | 29·75 | 57 | 55 | WNW | W | Fine | | |
| 29 | | ‘10 | 43 | 56 | 38 | 29·47 | 29·33 | 60 | 57 | S | W | Showery | Rain | Fine |
| 30 | | | 41 | 51 | 36 | 29·57 | 29·68 | 57 | 58 | WSW | SSW | Fine | | |
| 31 | | ‘02 | 43 | 50 | 44 | 29·50 | 29·46 | 58 | 60 | SE | W | Fog | Cloudy | |
| Nov | | | | | | | | | | | | | | |
| 1 | | ‘03 | 48 | 51 | 36 | 29·41 | 29·63 | 60 | 60 | NW | WNW | Cloudy | Rain | Cloudy |
| 2 | | | 39 | 47 | 34 | 29·79 | 29·84 | 60 | 57 | W | W | Fine | | |
| 3 | | | 37 | 43 | 31 | 29·88 | 29·91 | 56 | 58 | W | W | Fog | | |
| 4 | | | 35 | 46 | 30 | 29·90 | 29·96 | 57 | 59 | E | NE | Cloudy | | |
| 5 | ● | ‘09 | 36 | 50 | 47 | 29·95 | 29·70 | 57 | 62 | NNE | SW | Fog | Cloudy | Rain |
| 6 | | ‘11 | 50 | 53 | 45 | 29.78 | 29·80 | 65 | 62 | W | W | Rain | Cloudy | Cloudy |
| 7 | | | 54 | 57 | 50 | 29·78 | 29·86 | 65 | 65 | WSW | SSE | Cloudy | | |
| 8 | | | 54 | 57 | 49 | 29·86 | 29·91 | 66 | 61 | E | E | Cloudy | | |
| 9 | | | 55 | 59 | 45 | 29·94 | 29·98 | 60 | 56 | ENE | ENE | Cloudy | | |
| 10 | | | 50 | 53 | 39 | 30·01 | 30·06 | 58 | 59 | NE | NNE | Fine | | Cloudy |
| 11 | | | 43 | 50 | 48 | 30·00 | 29·91 | 55 | 57 | NE | E | Fine | | |
| 12 | | ‘12 | 48 | 51 | 46 | 29·83 | 29.77 | 56 | 59 | W | WSW | Cloudy | | Rain |
| 13 | ☾ | ‘17 | 48 | 47 | 33 | 29·59 | 29·67 | 61 | 61 | E | ENE | Rain | | Cloudy |
| 14 | | ‘11 | 38 | 44 | 31 | 29·81 | 29·82 | 59 | 58 | NE | NE | Cloudy | | Rain |
| 15 | | | 34 | 41 | 31 | 29·90 | 30·00 | 57 | 57 | NE | NNE | Fine | | |
| 16 | | | 35 | 40 | 31 | 29·93 | 29·85 | 57 | 55 | N | N | Fine | | |
| 17 | | ‘18 | 34 | 41 | 35 | 29·61 | 29·57 | 58 | 62 | SW | WSW | Cloudy | Rain | |
| 18 | | | 38 | 45 | 33 | 29·86 | 29·81 | 60 | 60 | WNW | W | Fog | | |
| 19 | | ‘01 | 36 | 47 | 39 | 29·9[illegible] | 30·00 | 61 | 60 | WNW | W | Fog | | Fine |

The quantity of rain fallen in the month of October,
is 1 inch and 81-100ths.

### REPORT OF DISEASES.

OUR several Reports present nothing particularly remarkable in the diseases of the past month. Pulmonary disorders, rheumatism, and scarlatina,—which has been transmitted, since our last Report, from children to adults, to some considerable extent,—and measles, are the most prevalent affections: the last has shown, perhaps, an unusual

4

disposition to give rise, on its termination, to diarrhœa, from irritation of the mucous membrane of the intestines, in children. Whooping-cough is very common; but it has not shown much severity in the generality of cases. A remarkable case of hydrencephalus occurred in a child three years old, subsequent to an attack of partial tetanus, without obvious cause, in which the head and neck were bent backwards, of a few days' duration. The anterior fontanel was open when the child was seen by the Reporter, and fluctuation of a fluid could be perceived beneath the integuments. It terminated fatally. Permission to examine the body could not be obtained. Much temporary alleviation was derived from a bandage placed round the head firmly, as recommended by Sir Gilbert Blane: when it was removed for a minute or two, the child's sufferings were obviously increased, and strongly marked in its countenance. Binding up the head for every kind of head-ache, is a regular practice amongst the common people of Wales and Brittany, and it is said by them to be often used with success.

Small-pox is hardly to be met with. It is an extraordinary circumstance that it did not prevail to a greater extent a few months since, when it was seen in several parts of London in a very severe form; for it is probable that about one-half of the children of the metropolis above a year old, amongst the lower classes of society, are unprotected from its influence. Thus, amongst fifty such children which have claimed our assistance, for various diseases, within a few weeks, only fourteen have had cow-pox; six have had small-pox from inoculation, and ten took this disease naturally. This number is too small to permit any calculations from it being generally applied; but we shall notice this point again hereafter. In the mean time, these observations may lead some of our readers to be more active in their efforts to overcome the prejudices of the lower classes against vaccination.

A considerable number of the cases of acute rheumatism mentioned in our last Report, have passed into the chronic form; while many of those habitually subject to this disease have suffered new attacks during the late very variable state and temperature of the atmosphere.

Since the weather became foggy, dyspnœa has become a more general and urgent symptom in the chronic pectoral complaints of old people, and some instances of spasmodic asthma have occurred. Many cases of phthisis pulmonalis, which had advanced but very slowly for some months, are now hastening rapidly to a close. Erysipelas, principally affecting the head and face, has made its appearance. Without offering an opinion on the practice of treating this disease with bark, so very generally adopted in our hospitals, we may be allowed to remark, that, out of a considerable number of patients treated with antimonials and purgatives, and the topical use of cold spirituous lotions, we have not seen the disease in any instance fatal. A few, and but a few, instances of typhus have occurred; but they have not presented any peculiarity worthy of remark.

